# The Influence of Robot Facial Expression and Inclusion Behavior on Rapport and Trust in Older Adults: Mixed-Factorial Experimental Study

**DOI:** 10.2196/91235

**Published:** 2026-07-15

**Authors:** Hsiu-Ping Yueh, Kuang-Chi Lin, Weijane Lin

**Affiliations:** 1 Department of Psychology/Bio-Industry Communication & Development/Mechanical Engineering, Research Center for Digital Humanities, National Taiwan University, Taipei, Taiwan; 2 Research Center for Digital Humanities, National Taiwan University, Taipei, Taiwan; 3 Department of Library and Information Science, Research Center for Digital Humanities, National Taiwan University, No. 1, Sec. 4, Roosevelt Rd., Daan Dist., Taipei, Taiwan, 106319, Taiwan, 886 233662971

**Keywords:** social robots, human-robot interaction, rapport, facial expression, healthy aging

## Abstract

**Background:**

The World Health Organization framework for healthy aging emphasizes that the capacity to establish and sustain relationships is a vital component of functional ability. Social robots offer valuable support for this relational aspect, although their effectiveness is contingent upon the quality of the interactions. While it is established that facial expressions and inclusive behaviors can influence rapport building, the combined effects of these elements on older adults remain unexamined.

**Objective:**

This study aimed to investigate the main and interaction effects of robot facial expression (happy vs unhappy) and interaction style (inclusive vs exclusive) on the development of rapport between social robots and older adults residing in the community.

**Methods:**

A 2 × 2 mixed-factorial experimental design was used in this study, involving 53 community-dwelling older adults (mean age 70.57, SD 4.27 years; n=45, 84.9% female). Participants engaged in a gamified interaction known as Radish Squat featuring 2 humanoid robots that exhibited various combinations of facial expressions and interaction styles. The inclusive robot designated participants within the game 75% of the time, whereas the exclusive robot did so approximately 25% of the time. Dependent measures included robot acceptance, rapport, rapport expectation, and trust. Behavioral designation frequencies were recorded throughout the gameplay. A mixed-design repeated-measure ANOVA was conducted to examine both main and interaction effects.

**Results:**

Facial expression exhibited significant main effects on acceptance (*F*_1,51_=14.27; *P*<.001; η^2^=0.22), rapport (*F*_1,51_=6.69; *P*=.01; η^2^=0.12), conversation partner expectations (*F*_1,51_=11.34; *P*=.001; η^2^=0.18), togetherness expectations (*F*_1,51_=10.28; *P*=.002; η^2^=0.17), and trust (*F*_1,51_=4.36; *P*=.04; η^2^=0.08). Interaction style significantly affected rapport (*F*_1,51_=6.22; *P*=.02; η^2^=0.11), as well as both rapport expectation subscales. Significant interaction effects were also observed for rapport (*F*_1,51_=8.44; *P*=.005; η^2^=0.14) and trust (*F*_1,51_=16.62; *P*=.002; η^2^=0.25) such that the positive effect of happy facial expressions was substantially larger under exclusive interaction conditions. Older adults designated the exclusive robot within the game more frequently than the inclusive robot (mean 6.42, SD 1.84 vs 5.32, SD 2.12; *t*_52_=2.18; *P*=.03), and qualitative data pointed to the use of fairness-based turn-taking strategies.

**Conclusions:**

Positive facial expressions and inclusive interaction behaviors significantly enhanced rapport and trust, with synergistic interaction effects primarily emerging under exclusive interaction conditions. The observed patterns, whereby older adults compensated for exclusive robot behavior, suggest that they perceive robots as social entities deserving of equitable treatment. Collectively, these findings offer evidence-based guidelines for designing social robots that foster the relational dimensions of healthy aging.

## Introduction

### Background

The aging global population necessitates a fundamental shift in our approach to supporting the well-being of older adults. By 2030, it is projected that 1 in 6 individuals worldwide will be aged 60 years or older, and this number is projected to reach 2.1 billion by 2050. In most high-income nations, the age of 65 years is widely accepted as the conventional threshold for retirement and is commonly adopted as a criterion for defining the older adult demographic [1-3]. Accordingly, the World Health Organization has defined healthy aging not simply as the absence of disease but as “the process of developing and maintaining the functional ability that enables well-being in older age” [4-6]. This functional ability, comprising what individuals value in terms of being and doing, emerges from the dynamic interplay between their intrinsic capacity and environmental factors [4,5]. Of the 5 essential domains of functional ability, the capacity to build and maintain relationships is particularly important as social connections profoundly impact stress regulation, cognitive function, and overall well-being [1,6]. However, aging often precipitates social network contraction due to factors such as spousal loss, family dispersion, and mobility limitations. Notably, social isolation has been identified as a mortality risk comparable to smoking [7]. This relational deficit underscores the urgent need for environmental interventions that offer accessible and consistent opportunities for positive social interaction.

Social robots represent a promising environmental resource for addressing this relational dimension of healthy aging. Meta-analytic evidence demonstrates that robot interventions can effectively reduce loneliness and improve psychological well-being among older adults [8,9]. However, the successful integration of social robots into older adults’ lives depends critically on interaction quality rather than mere functional utility [10,11]. The computers are social actors (CASA) paradigm establishes that humans unconsciously apply social rules to technological agents, expecting adherence to norms of politeness, turn taking, and emotional congruence [12,13]. For older adults with deeply established social schemas, these expectations are particularly pronounced [11,14]. When robots fail to meet social expectations, the resulting dissonance undermines potential benefits. Therefore, social robots must be conceptualized not as passive tools but as active environmental components whose design features determine whether they enable or hinder the relational aspects of functional ability [14,15].

Rapport, a harmonious relationship characterized by mutual attentiveness, positivity, and coordination [16], emerges as the critical mechanism linking robot design to healthy aging outcomes. When rapport is established, older adults experience enhanced engagement, increased motivation for continued interaction, and greater trust in robot capabilities [17,18]. Research has identified nonverbal cues as primary drivers of rapport formation, with facial expressions serving as powerful signals of social intent [19,20]. Additionally, social inclusion behaviors constitute a fundamental dimension: studies adapting the Cyberball paradigm demonstrate that humans respond to robot-mediated inclusion and exclusion similarly to human interactions [21,22]. However, a critical gap remains. While recent research with university students revealed complex patterns when facial expressions and interaction styles were manipulated simultaneously [23], it remains uncertain how older adults process these potentially conflicting cues. An incongruent robot, one that displays positive expressions while behaving in an exclusive manner, may generate mistrust rather than rapport. Given the distinct generational experiences and varying intrinsic capabilities of older adults [24], understanding the combined effects of these rapport-related characteristics is essential for designing robots that genuinely support healthy aging.

### Study Objectives

Driven by the previously mentioned challenges, this study examined the impact of robot facial expressions (happy vs unhappy) and interaction styles (inclusive vs exclusive) on rapport building with community-dwelling older adults (aged 65 years or older). Using a gamified interaction paradigm, we focused on (1) the main effects of facial expressions and interaction styles on acceptance, rapport, rapport expectations, and trust; (2) the interaction effects of these 2 characteristics; and (3) the behavioral patterns exhibited by older adults during gamified human-robot interaction (HRI). By analyzing how older adults respond to both congruent and incongruent combinations of robot social cues, this research offers evidence-based guidelines for designing social robots that effectively support healthy aging.

## Methods

### Study Design

This study used a mixed-factorial experimental design with a 2 (facial expression: happy vs unhappy) × 2 (interaction style: inclusive vs exclusive) structure. Experimental condition (congruent vs incongruent assignment) served as the between-subject factor, whereas facial expression and interaction style were manipulated within participants. During task 2, each participant interacted with both robots, thereby enabling direct within-subject comparisons. This design allowed for the examination of both main effects and interaction effects. Participants were randomly assigned to 1 of 2 experimental conditions.

In the congruent condition (group 1), robot 1 exhibited a happy facial expression and inclusive interaction behavior, whereas robot 2 exhibited an unhappy facial expression and exclusive interaction behavior. In the incongruent condition (group 2), robot 1 exhibited an unhappy facial expression with inclusive interaction behavior, whereas robot 2 exhibited a happy facial expression with exclusive interaction behavior.

### Ethical Considerations

This study was reviewed and approved by the Research Ethics Committee of National Taiwan University (202305HS096). All participants provided informed consent before taking part. All collected data were deidentified and stored securely. Participants received NTD 200 (NTD $1=US $0.03 as of July 6, 2026) as compensation for their participation. Upon completion of all measures and the postexperiment interview, participants received a full debriefing that explained the experimental manipulation and clarified that the exclusive robot’s behavior was experimentally scripted rather than contingent on the participants.

### Participants

Community-dwelling older adults were recruited through volunteer networks at the university and through snowball sampling. The following inclusion criteria were established for participants: (1) age of 65 years or above, (2) normal cognitive function and hearing ability, (3) fluency in Mandarin to facilitate verbal interactions with robots, (4) normal or corrected vision adequate for perceiving robot facial expressions at a conversational distance, and (5) sufficient upper-body mobility to engage in game actions.

The sample comprised 53 participants, whose characteristics and experiences with robot interaction are detailed in Table 1. The average age of the participants was 70.57 (SD 4.27; range 65-85) years. The substantial majority (n=45, 84.9% of the participants) identified as female. Educational attainment varied among the group, with the largest proportion holding university degrees (n=22, 41.5%). Furthermore, most participants reported limited experience with robots, interacting with them less than once in the previous 6 months (n=44, 83%). Independent-sample 2-tailed *t* tests and chi-square tests indicated no significant differences between group 1 and group 2 on any baseline characteristics (*P*>.10 in all cases), supporting the success of the randomization procedure.

**Table 1.** Demographics and experiences of the participants (N=53).

Characteristic	Total	Group 1^a^ (n=27)	Group 2^b^ (n=26)
Age (years), mean (SD)	70.57 (4.27)	70.67 (4.79)	70.46 (3.76)
Age range (years)	65-85	65-85	65-79
Age group (years), n (%)			
65-69	24 (45.3)	13 (48.1)	11 (42.3)
≥70	29 (54.7)	14 (51.9)	15 (57.7)
Sex, n (%)			
Female	45 (84.9)	23 (85.2)	22 (84.6)
Male	8 (15.1)	4 (14.8)	4 (15.4)
Educational level, n (%)			
Junior high school	3 (5.7)	2 (7.4)	1 (3.8)
High school	18 (34)	8 (29.6)	10 (38.5)
University or college	22 (41.5)	13 (48.1)	9 (34.6)
Graduate school	10 (18.9)	4 (14.8)	6 (23.1)
Personality, mean (SD)			
Extraversion (TIPI^c^ [2-14])	8.42 (2.26)	8.48 (2.31)	8.35 (2.24)
Empathy, mean (SD)			
Cognitive empathy (12-29)	21.04 (7.10)	21.07 (9.07)	21 (4.41)
Emotional contagion (22-40)	28.96 (3.31)	28.22 (2.93)	29.73 (3.55)
Robot contact frequency			
Less than once in the previous 6 mo, n (%)	44 (83)	25 (92.6)	19 (73.1)
Robot acceptance score^d^, mean (SD)	35.06 (3.93)	34.04 (4.31)	36.12 (3.24)
Robot attitude, mean (SD)			
NARS^e^ score	34.09 (9.80)	35.26 (9.88)	32.88 (9.77)
HRI^f^ belief, mean (SD)			
SE-HRI^g^ score	46.42 (7.53)	45.85 (6.60)	47 (8.48)

a Congruent condition (expression matched behavior).

b Incongruent condition (expression was mismatched with behavior).

c TIPI: Ten-Item Personality Inventory.

d Robot acceptance measured as total score (range 8-40).

e NARS: Negative Attitudes Toward Robots Scale (range 14-78; higher scores indicate more negative attitudes).

f HRI: human-robot interaction.

g SE-HRI: Self-Efficacy in Human-Robot Interaction Scale (range 10-60; higher scores indicate greater self-efficacy).

### Apparatus

**Figure 1.**
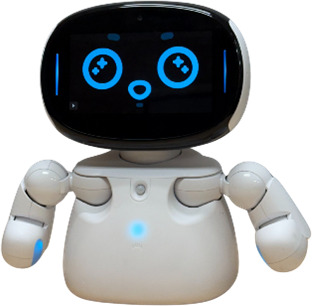
Kebbi robot used in this study.

The study used Kebbi humanoid robots produced by NUWA Robotics (Figure 1). Each Kebbi robot stands at approximately 35 cm tall and is equipped with a wheeled base for mobility, articulated arms and elbows, and a touch screen face capable of conveying a wide range of emotions. In total, 3 Kebbi robots were used: 1 served as the neutral expression model for practice (designated as R0), whereas the other 2 participated in the main experimental task (referred to as R1 and R2). The robots were strategically positioned on a tabletop to ensure they were at eye level with the seated participants. Their behaviors were manipulated through the Wizard of Oz technique combined with the Message Queuing Telemetry Transport protocol to enable semiautonomous communication between the human users and the 2 robots.

### Experimental Task: Radish Squat Game

The experimental task was an adapted version of the Radish Squat game, which closely resembles the Cyberball paradigm used in research on social exclusion [22,23]. In this game, players take turns executing specific actions while verbally stating their identity and designating the next player. Actions are categorized into 2 groups: basic actions (raising a hand, nodding, and shaking the head) and advanced actions (flapping wings, hugging, and drumming). This format facilitates natural, repeated interactions while systematically controlling the dynamics of inclusion and exclusion.

### Manipulation of Independent Variables

#### Facial Expression

**Figure 2.**
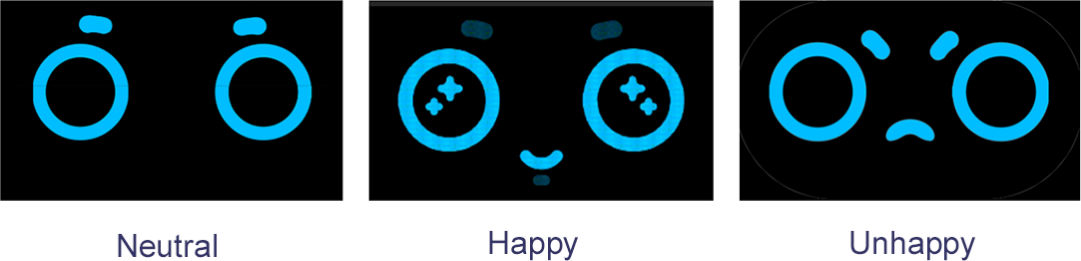
Robot facial expression designs.

The robot’s facial expressions were developed based on the elements associated with rapport as outlined by Wang and Gratch [20]. As depicted in Figure 2, the happy expression featured upturned corners of the mouth and a lifted chin, whereas the unhappy expression was characterized by furrowed brows, a wrinkled nose, and a raised upper lip. In the practice session, a neutral expression was used.

#### Interaction Style

In accordance with established protocols [21,23], the inclusive robot designated participants 75% of the time while allocating 25% of the time to interaction with the other robot. Conversely, the exclusive robot designated participants only 25% of the time, dedicating the remaining 75% of the time to interaction with the other robot.

### Procedure

**Figure 3.**
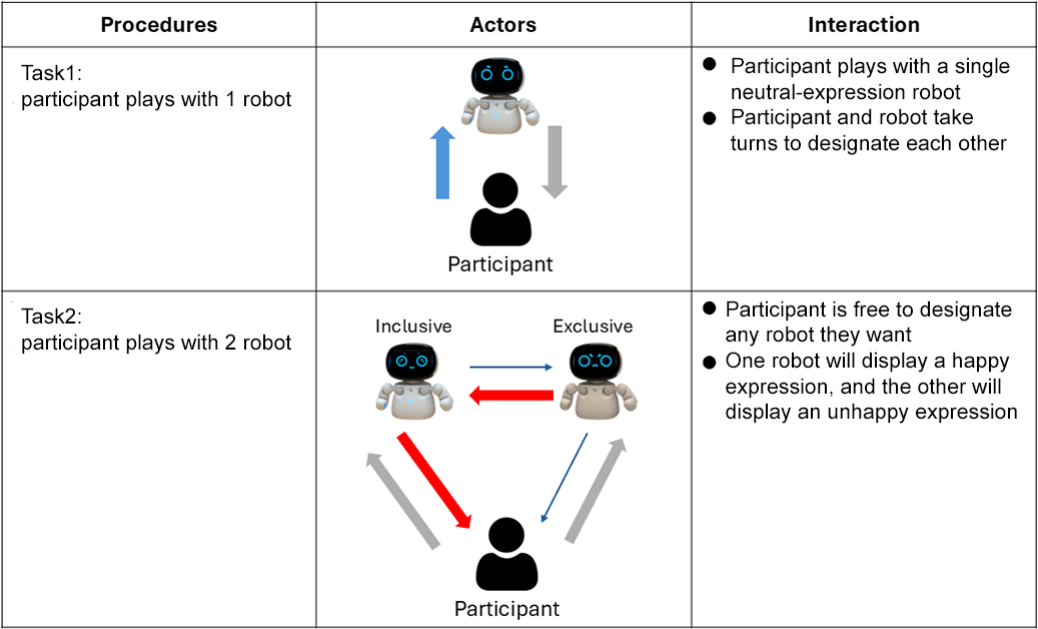
Experimental procedure flowchart.

The experiment consisted of 2 tasks (Figure 3). In task 1 (practice), participants engaged in the Radish Squat game with a single robot (R0) displaying a neutral facial expression for 12 turns to familiarize themselves with the task rules. In task 2 (main session), participants interacted simultaneously with 2 robots (R1 and R2), each exhibiting a distinct combination of facial expression and interaction style, over a total of 30 turns. The task 2 Radish Squat session lasted for an average of 12.4 (SD 2.1; range 9-17) minutes. Following task 2, participants completed postexperimental questionnaires and took part in brief semistructured interviews. The entire experimental session lasted approximately 45 minutes.

### Measures

Four dependent variables were assessed following interaction with each robot: robot acceptance, rapport, rapport expectation, and trust. Robot acceptance was measured using 8 items based on the technology acceptance model ([23,25]; item score range 1-5; higher scores indicate greater acceptance). Rapport was assessed using a 9-item rapport scale ([17]; Cronbach α=0.85; item score range 1-5; higher scores indicate greater rapport). Rapport expectation was measured using the 18-item Rapport-Expectation With a Robot Scale [26], comprising 2 subscales: expectation as a conversation partner (11 items; Cronbach α=0.85) and expectation for togetherness (7 items; Cronbach α=0.82; item score range 1-7; higher scores indicate greater expectation). Trust was assessed using the 15-item Trust Perception Scale–HRI ([27]; item score range 1-6; higher scores indicate greater trust). Behavioral data were collected as the number of times participants designated each robot during gameplay. Baseline measures included extraversion (Ten-Item Personality Inventory [28]), empathy (Basic Empathy Scale [29]), negative attitudes toward robots (Negative Attitudes Toward Robots Scale [30]), and self-efficacy in HRI (Self-Efficacy in Human-Robot Interaction Scale [31]). Brief semistructured interviews were conducted after the experiment to gather qualitative insights regarding participants’ interaction experiences and observations of robot behaviors. Table 2 summarizes all measures used in this study.

**Table 2.** Summary of measures.

Category and measure	Items, n	Scale	Reference
Dependent			
Robot acceptance	8	5-point	[23,25]
Rapport	9	5-point	[17]
Rapport expectation—conversation partner	11	7-point	[26]
Rapport expectation—togetherness	7	7-point	[26]
Trust	15	6-point	[27]
Designation frequency	—^a^	—	[22,23]
Baseline			
Extraversion (TIPI^b^)	2	7-point	[28]
Basic empathy	20	5-point	[29]
NARS^c^	14	5-point	[30]
SE-HRI^d^	10	6-point	[31]
Qualitative			
Postinteraction interview	—	—	[22,23]

a Not applicable.

b TIPI: Ten-Item Personality Inventory.

c NARS: Negative Attitudes Toward Robots Scale.

d SE-HRI: Self-Efficacy in Human-Robot Interaction Scale.

### Statistical Analysis

A mixed-design repeated-measure ANOVA was conducted with facial expression (happy vs unhappy) and interaction style (inclusive vs exclusive) as within-subject factors and experimental condition (congruent vs incongruent) as the between-subject factor. Both main effects and interaction effects were analyzed for each dependent variable. Effect sizes were reported as partial η^2^ and interpreted using the benchmarks by Cohen ([32]; small: η^2^≥0.01; medium: η^2^≥0.06; large: η^2^≥0.1). Post hoc power analyses (α=.05) indicated adequate power for the detected interaction effects: for trust (η^2^=0.25), achieved power was approximately 0.97; for rapport (η^2^=0.14), power was approximately 0.83. Post hoc comparisons were performed using the Bonferroni correction. Additionally, paired 2-tailed *t* tests were used to compare the frequencies of behavioral designations between the robots (*t*_52_=2.18; *P*=.03).

## Results

### Manipulation Check

To conduct the postexperimental manipulation checks, each participant was interviewed to verify the successful implementation of both independent variables. With respect to facial expressions, chi-square tests indicated that participants correctly identified the happy-expression robot as displaying a happy expression at rates significantly above chance (N=53, *χ*^2^_1_=38.4; *P*<.001) and the unhappy-expression robot as displaying an unhappy expression at rates significantly above chance (N=53, *χ*^2^_1_=31.7; *P*<.001). Additionally, as shown in Table 3, the inclusive robot engaged participants significantly more frequently than the exclusive robot, further validating the behavioral manipulation.

**Table 3.** Frequency of robot designation by interaction style: manipulation check^a^.

Robot type	Designations^b^, mean (SD)
Inclusive	8.77 (1.73)
Exclusive	2.79 (1.79)

a *t*_52_=24.31; *P*<.001.

b Designations refer to the number of times each robot designated the participants during task 2.

### Descriptive Statistics

Table 4 presents descriptive statistics for all dependent variables across the 4 within-subject experimental conditions. The patterns were more nuanced than a simple summary of main effects would suggest. For acceptance, robots with happy expressions received higher ratings than those with unhappy expressions across both interaction styles, aligned with their high robot acceptance and HRI belief (Table 1). For rapport and trust, however, the advantage of happy expressions was concentrated primarily in the exclusive interaction condition, with only minimal expression-related differences under inclusive conditions. These patterns are elaborated on in the interaction analysis reported below.

**Table 4.** Mean scores of dependent variables by experimental condition.

Variable	Inclusive, mean (SD)	Exclusive, mean (SD)	Overall, mean (SD)
Acceptance (1-5)			
Happy expression	4.25 (0.54)	4.65 (0.41)	4.45 (0.52)
Unhappy expression	4.17 (0.65)	4.08 (0.74)	4.12 (0.689)
Rapport (1-5)			
Happy expression	3.74 (0.62)	4.44 (0.49)	4.08 (0.66)
Unhappy expression	3.88 (0.79)	3.77 (0.85)	3.82 (0.81)
Expectations as a conversation partner (1-7)			
Happy expression	4.33 (0.56)	4.85 (0.77)	4.58 (0.72)
Unhappy expression	4.41 (0.66)	4.01 (0.85)	4.20 (0.79)
Expectations for togetherness (1-7)			
Happy expression	4.81 (0.67)	5.65 (0.78)	5.22 (0.83)
Unhappy expression	5.07 (0.90)	4.54 (1.20)	4.80 (1.09)
Trust (1-6)			
Happy expression	4.48 (0.65)	5.13 (0.47)	4.79 (0.65)
Unhappy expression	4.55 (0.72)	4.66 (0.77)	4.61 (0.74)

### Main Effects and Interaction Effects

#### Overview

Table 5 presents the results of the repeated-measure ANOVA. Facial expression demonstrated significant main effects across all 5 outcome measures, with effect sizes ranging from small to large. Interaction style significantly influenced rapport and both rapport expectation subscales. Significant interaction effects were observed for rapport and trust (both large effects), indicating that the combined influence of facial expression and interaction style was more complex than a simple additive pattern.

**Table 5.** Results of repeated-measure ANOVA: main effects and interaction effects^a^.

Variable	Facial expression			Interaction style			F × I^b^	
	*F* test (*df*)	*P* value	η^2^	*F* test (*df*)	*P* value	η^2^	*F* test (*df*)	η^2^
Acceptance	14.27 (1, 51)	<.001	0.22	3.18 (1, 51)	.08	0.06	3.41 (1, 51)	0.06
Rapport	6.69 (1, 51)	.01	0.12	6.22 (1, 51)	.02	0.11	8.44 (1, 51)^c^	0.14
Expectations as a conversation partner	11.34 (1, 51)	.001	0.18	7.99 (1, 51)	.007	0.14	0.29 (1, 51)	0.01
Expectations for togetherness	10.28 (1, 51)	.002	0.17	10.47 (1, 51)	.002	0.16	0.41 (1, 51)	0.01
Trust	4.36 (1, 51)	.04	0.08	2.91 (1, 51)	.10	0.05	16.62 (1, 51)^c^	0.25

a Effect size benchmarks [32]: η2≥0.01 for small, η2≥0.06 for medium, and η2≥0.1 for large.

b Facial expression × interaction style.

c *P*<.01*.*

#### Acceptance

**Figure 4.**
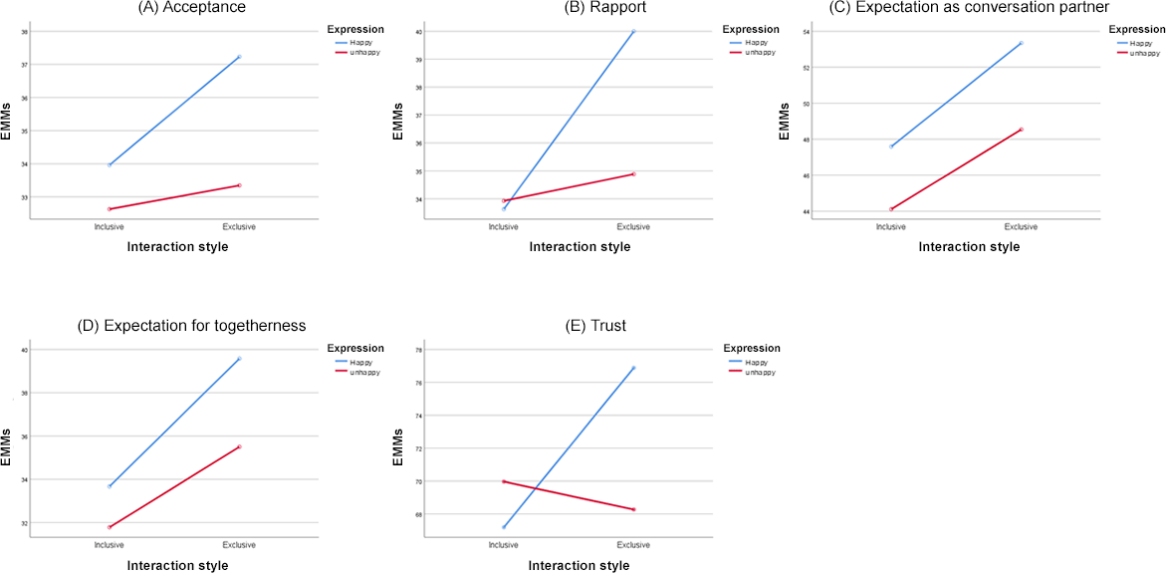
Interaction effects of facial expression and interaction style. EMM: estimated marginal mean.

Facial expression had a significant main effect on acceptance (*F*_1,51_=14.27; *P*<.001; η^2^=0.22), with robots displaying happy expressions receiving higher ratings (mean 4.45, SD 0.52) than those displaying unhappy expressions (mean 4.12, SD 0.689). As shown in Figure 4A, the main effect of interaction style was not significant (*F*_1,51_=3.18; *P*=.08; η^2^=0.06), nor was the interaction between facial expression and interaction style (*F*_1,51_=3.41; *P*=.07; η^2^=0.06). In the absence of a significant interaction, the main effect of facial expression can therefore be interpreted directly.

#### Rapport

For rapport, a significant interaction effect was observed (*F*_1,51_=8.44; *P*=.005; η^2^=0.14). In light of this interaction, the main effects were qualified and interpreted via simple effects. As shown in Figure 4A, 4B, and 4C, under exclusive interaction, the happy-expression robot generated substantially higher rapport (mean 4.44, SD 0.49) than the unhappy-expression robot (mean 3.77, SD 0.85). In contrast, under inclusive interaction, there was minimal difference between happy (mean 3.74, SD 0.62) and unhappy (mean 3.88, SD 0.79) expressions, and this difference was not significant following Bonferroni correction (*P*=.65). Thus, the advantage of happy facial expressions was concentrated in the exclusive condition. Accordingly, the overall main effect of facial expression (*F*_1,51_=6.69; *P*=.01; η^2^=0.12) and the main effect of interaction style (*F*_1,51_=6.22; *P*=.02; η^2^=0.11) should be understood as being driven by this asymmetry rather than uniform effects across interaction conditions.

#### Rapport Expectation

Regarding expectations for a conversation partner, both facial expression (*F*_1,51_=11.34; *P*=.001; η^2^=0.18) and interaction style (*F*_1,51_=7.99; *P*=.007; η^2^=0.14) exhibited significant main effects, whereas their interaction was not significant (Figure 4C; *F*_1,51_=0.29; *P*=.59; η^2^=0.01). As shown in Table 4, robots with happy expressions elicited higher conversation partner expectations (overall mean 4.58, SD 0.72) than those with unhappy expressions (mean 4.20, SD 0.79), and exclusive robots elicited higher expectations (marginal mean 4.43) than inclusive robots (marginal mean 4.37). In the absence of a significant interaction, these main effects can be interpreted directly.

For togetherness expectations, both facial expression (*F*_1,51_=10.28; *P*=.002; η^2^=0.17) and interaction style (*F*_1,51_=10.47; *P*=.002; η^2^=0.16) yielded significant main effects, with no significant interaction (*F*_1,51_=0.41; *P*=.52; η^2^=0.01). Robots with happy expressions elicited higher togetherness expectations (overall mean 5.22, SD 0.83) than those with unhappy expressions (mean 4.80, SD 1.09). As shown in Figure 4D, although inclusive robots tended to elicit lower togetherness expectations than exclusive robots within the happy-expression condition, this pattern did not reach the level of significant interaction.

#### Trust

For trust, a significant interaction effect was observed (*F*_1,51_=16.62; *P*=.002; η^2^=0.25). As shown in Figure 4E, under exclusive interaction, the happy-expression robot received substantially higher trust ratings (mean 5.13, SD 0.47). Under inclusive interaction, the pattern was slightly reversed: trust ratings for the happy-expression (mean 4.48, SD 0.65) and unhappy-expression (mean 4.55, SD 0.72) robots were essentially equivalent, and this difference was not significant following Bonferroni correction. The significant main effect of facial expression (*F*_1,51_=4.36; *P*=.04; η^2^=0.08), therefore, reflects the strong expression effect in the exclusive condition. The main effect of interaction style was not significant overall (*F*_1,51_=2.91; *P*=.10; η^2^=0.05).

#### Behavioral Patterns: Designation Frequency

The analysis of behavioral data unveiled an unexpected pattern in the designation behavior of participants (Table 6). Contrary to established expectations, participants designated the exclusive robot significantly more frequently than the inclusive robot. This finding stands in contrast to earlier research with university students, who tended to favor the inclusive robot [23]. Qualitative interview data suggested that participants’ designation behaviors were guided primarily by considerations of fairness and turn taking.

**Table 6.** Participants’ designation frequency during task 2^a^.

Target robot	Designations, mean (SD)	Total designations, n
Inclusive interaction	5.32 (2.12)	282
Exclusive interaction	6.42 (1.84)	340
Happy expression	6.09 (2.06)	323
Unhappy expression	5.64 (2.04)	299

a Inclusive vs exclusive: *t_52_*=2.18 (*P*<.05); happy vs unhappy: *t*_52_=0.87.

Representative interview quotes illustrating fairness-based designation strategies included the following:

I wanted to ensure fair turn-taking so everyone could participate.

[G1-17-F]

Since the blue robot kept choosing the red robot, I designated blue to make it fair.

[G2-16-F]

I simply picked them in turns to give everyone a chance to play.

[G1-4-F]

I assigned both robots because I believe the game should involve taking turns.

[G2-1-M]

I chose randomly since we were playing together and wanted everyone to have their turn.

[G1-26-M]

These responses suggest that older adults actively sought to balance the exclusive robot’s behavior. They consistently used turn-taking strategies to maintain fairness and equitable participation, including for the robots themselves.

## Discussion

### Principal Findings

This study examined how a robot’s facial expressions and interaction style affect the development of rapport between older adults and social robots. Our findings revealed that (1) positive facial expressions significantly enhanced acceptance, rapport, rapport expectations, and trust; (2) an inclusive interaction style contributed to improved rapport and heightened rapport expectations; (3) there were notable interaction effects between facial expression and interaction style on both rapport and trust; and (4) older adults demonstrated a strong tendency toward inclusivity regarding robots, actively compensating for any behaviors that may seem exclusive.

### Comparison With Prior Work

Our findings regarding the impact of facial expressions are consistent with those of prior research indicating that positive robot expressions enhance the quality of interactions [19,33]. The observed effect sizes (η^2^=0.08-0.22) point to medium to large effects, underscoring the importance of facial expression as a significant cue for older adults in rapport building.

The interaction effects between facial expression and interaction style represent a novel theoretical contribution. Under inclusive conditions, the robot’s behavior reliably signaled social engagement and satisfied CASA-derived expectations for turn taking and politeness norms [12,13]. This behavioral sufficiency may attenuate reliance on facial expression as a diagnostic cue. Under exclusive conditions, however, the behavioral signal was ambiguous or negative, creating a cue vacuum that heightened the diagnostic value of facial expressions as participants sought to infer the robot’s social intentionality. The large interaction effect for trust (η^2^=0.25) is particularly noteworthy: trust in the exclusive robot was substantially modulated by facial expression, whereas trust in the inclusive robot remained relatively stable regardless of expression. This pattern aligns with the extension of CASA by Gambino et al [12], which posits that, when behavioral signals deviate from social norms, people rely more heavily on secondary cues such as appearance. Incongruent combinations (positive expression+exclusive behavior) appeared to intensify attention to facial cues as participants attempted to interpret the robot’s intentions, a process that, in this sample, ultimately yielded higher rapport with and trust in the happy-faced exclusive robot, suggesting that positive expression may partially compensate for exclusive behavior [23]. Future research using mediation designs could formally test the proposed attentional pathway.

Notably, older adults designated the exclusive robot more frequently than the inclusive robot, in stark contrast to university students in earlier research [23], who favored the inclusive robot. This generational difference may reflect divergent values related to fairness and group harmony. In the socioemotional selectivity theory by Carstensen [34], older adults prioritize emotionally meaningful relationships and prosocial goals. This orientation may lead them to perceive the exclusive robot as a social agent in need of remediation and actively compensate through equitable turn assignment. This pattern is also consistent with prosocial aging research documenting heightened concern for fairness among older adults [24,35]. Future studies using more gender-balanced samples would be valuable for determining whether this inclusivity tendency is particularly characteristic of older women, who constituted the majority of our sample.

### Implications for Healthy Aging Interventions

These findings carry important implications for the design of social robots that support healthy aging. First, positive facial expression capabilities appear essential as they produced medium to large enhancements across a range of rapport-related outcomes. Second, designers should prioritize inclusive interaction behaviors that actively engage older users. Third, the observed interaction effects indicate that optimizing individual features in isolation may not yield optimal combined outcomes. The mutual amplification of facial expression and interaction style, particularly in exclusive contexts, needs to be considered from a holistic design perspective.

From a public health standpoint, the demonstrated capacity of older adults to establish rapport with robots through brief, gamified interactions underscores the feasibility of scalable robot-mediated interventions. Moreover, the finding that older adults viewed robots as entities deserving of equitable treatment highlights the potential for meaningful human-robot relationships to complement human care in addressing loneliness and social isolation [36].

### Limitations

This study offers valuable observational insights; however, several limitations warrant careful consideration. First, our sample was predominantly female (45/53, 84.9%), likely reflecting recruitment through university-affiliated volunteer networks in Taiwan, which tend to overrepresent women among older adults. Because gender can influence social cue processing and trust building in HRI [30,37], the present findings may be more directly applicable to older women. Future studies with more gender-balanced or male-majority samples are needed to assess generalizability. Second, the gamified Radish Squat interaction enabled precise experimental control but may differ from naturalistic caregiving contexts in duration, emotional stakes, and task demands. The rapport captured in this study reflects initial formation in a brief, structured setting; whether these patterns persist and predict rapport stability in long-term caregiving relationships requires longitudinal investigation [38]. Nonetheless, gamified formats are increasingly adopted in health-promoting robot interventions for older adults, lending direct relevance to the present findings. Third, the single-session design precludes conclusions about long-term rapport development or the cumulative effects of repeated exposure. Additionally, our focus on healthy, community-dwelling adults suggests that the findings may not be applicable to individuals with cognitive impairments. Future research could benefit from systematically examining gender differences, using longitudinal designs, and investigating the cognitive and neurobiological mechanisms that may underpin older adults’ strong inclusivity responses toward robots.

### Conclusions

This study demonstrates that gamified interactions can effectively foster rapport between older adults and social robots. Positive facial expressions and inclusive interaction behaviors enhanced acceptance, rapport, rapport expectation, and trust. Crucially, these elements interacted synergically: the positive impact of happy facial expressions was substantially amplified when the robot behaved exclusively, suggesting a context-dependent cue-weighting process grounded in the CASA paradigm [12]. The unexpected finding that older adults compensated for exclusive robot behavior through fairness-based turn taking further suggests that they perceived these robots as social entities worthy of equitable treatment. Collectively, these insights offer evidence-based design guidelines for social robots intended to foster meaningful relationships with older adults and contribute to the development of technology-based interventions that promote healthy aging.

## Abbreviations

CASA: computers are social actors
HRI: human-robot interaction
